# Perspectives of using a digital test to support early detection of Alzheimer's: A protocol for involving underserved groups

**DOI:** 10.1002/alz70860_100753

**Published:** 2025-12-23

**Authors:** Sarah Wilson, Nehal Hassan, Dennis Chan, Sarah Slight

**Affiliations:** ^1^ Newcastle University, Newcastle Upon Tyne, United Kingdom; ^2^ University College London, London, London, United Kingdom

## Abstract

**Background:**

Alzheimer's Disease (AD) can develop up to 20 years before clinical symptoms become apparent. Spatial navigation is impaired in individuals at risk of AD and tests of this behaviour has higher diagnostic sensitivity and specificity for prodromal AD than current best‐in‐class cognitive tests.^(1,2)^ Spatial tests do not suffer from the same cultural and language confounds that affect legacy cognitive tests and are therefore better suited to diverse communities. As such, digital technologies measuring spatial behaviour may improve the early detection of AD in clinical practice in an inclusive manner. We have developed a protocol exploring the perspectives’ of underserved groups on using such digital tests as clinical diagnostic tools.

**Method:**

An inclusive recruitment process will be used to recruit underserved groups at risk of digital exclusion (defined in the CLEARS framework: Culture, Limiting Conditions, Education, Age, Residence, Socioeconomic status).^(3)^ Community organisations, which these groups may engage with, will be identified and time dedicated to building trusted relationships with gatekeepers. Recruitment sessions will be delivered at various community centres to build rapport with potential participants and understand their needs. Individuals interested in participating will complete a demographic questionnaire to assess eligibility and allow monitoring of equity and diversity. Consideration will be given to every eligible person regardless of socio‐demographic factors, disability and access. Invited participants will be asked to use the digital test in person followed by a semi‐structured interview to explore their perspectives. A framework thematic analysis approach, assisted by *N*‐Vivo, will be applied to the transcripts to identify key themes.

**Result:**

This qualitative study will recruit a diverse sample from underserved groups, identify facilitators and barriers to using the digital test, areas for improvement, and explore factors influencing acceptance of the test in clinical practice.

**Conclusion:**

The results of this study will be used to develop an inclusive digital tool that supports the early detection of AD. This protocol considers equality, diversity, and inclusion of participants at every stage.

Moodley, K., et al. (2015) *Hippocampus* 25: 939–51.

Ritchie, K., et al. (2018). JAD, 65(3), 885–896.

Wilson, S., et al. (2024). IJPP, 32(Supplement_1), i3‐i4.

